# Multi-heteroatom-doped porous carbon electrodes from 3D printing and conformal carbonization of ionic liquids for electrocatalytic CO_2_ conversion into syngas

**DOI:** 10.1038/s42004-025-01514-1

**Published:** 2025-04-23

**Authors:** Wei Wang, Na Zhao, Kai Zhao, Miao Zhang, Kanglei Pang, Yu Zhang, Jiayin Yuan

**Affiliations:** 1 School of Chemistry and Chemical Engineering, Lanzhou Jiaotong University, Lanzhou, China; 2 Department of Materials and Environmental Chemistry (MMK), Stockholm University, Stockholm, Sweden

**Keywords:** Electrocatalysis, Energy

## Abstract

3D printing as an advanced manufacturing technique provides an alternative cost-effective option for design and preparation of porous catalytic electrodes. Herein, carbonaceous catalytic electrodes with ternary dopants of boron (B), phosphorous (P), and nitrogen (N) (termed BPN-3Dp-CCEs) were successfully engineered *via* combination of the 3D printing technique and the following conformal carbonization of ionic liquid. The as-made electrodes were in turn applied to electrify CO_2_ into syngas in a controllable composition of a H_2_:CO molar ratio of 0.32–3.46. Notably, the BPN-3Dp-CCEs have tailored 3D macroscopic shapes of self-supporting skeletons, and due to ternary doping, demonstrated promoted catalytic activity in the electrocatalytic CO_2_ conversion into syngas. Upon optimization, the electrode remained stable in structure and performance after 10 h of a continuous CO_2_ electrolysis operation. This study casts insights and fuels the continuous exploration of multi-heteroatoms doped porous carbon electrodes for metal-free catalytic applications.

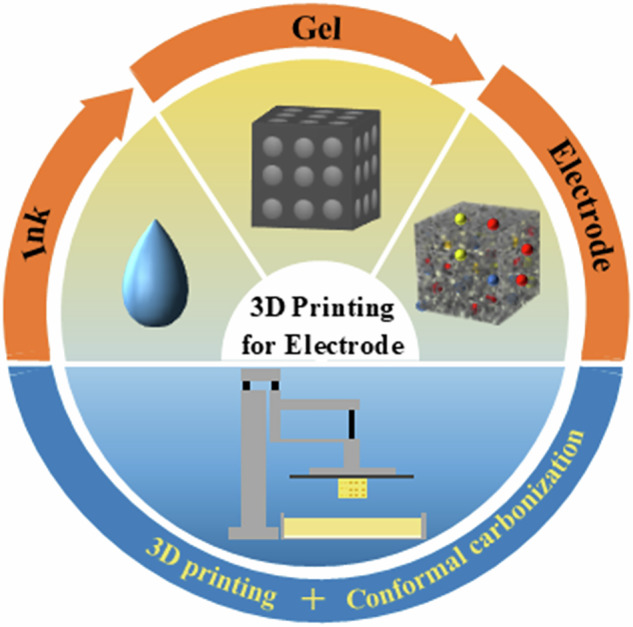

## Abstract (web summary)

3D printing offers significant advantages in creating customized electrode structures with enhanced catalytic performance. Here, the authors employ a combination of 3D printing and conformal carbonization of ionic liquids to engineer porous carbonaceous electrodes with boron, phosphorus and nitrogen dopants, achieving enhanced electrocatalytic activity for CO_2_ conversion into syngas with a controllable H_2_:CO molar ratio.

## Introduction

3D printing is a bottom-up, layer-by-layer revitalized manufacturing technique on the basis of digital model files^1,2^, and has attracted widespread interests^3,4^. It has been broadly used in diverse fields^5,6^, such as aerospace engineering, biomedical materials^7^, electronic devices, and electrochemical materials^8,9^. Typically, 3D printing offers distinct advantages as follows: (i) Its flexibility allows for tailored fabrication of electrode shapes, sizes, and patterns to meet specific requirements, thereby expanding their application scope. (ii) It enables precise control over porous structures, facilitating optimization of critical parameters such as porosity, surface area, and conductivity to enhance device performance. (iii) In comparison to electrodes preapred from powder-catalysts and glues, it typically exhibits higher structural integrity and stability for better durability and corrosion resistance. According to printing principles and molding methods, the 3D printing technique can be divided into several basic forms, such as materials extrusion, powder bed fusion, and photopolymerization. Notably, stereo lithography appearance (SLA) stands out of many 3D printing manufacturing methods. When it meets the preparation of catalytic electrodes, the self-supporting catalytic electrodes in 3D arbitrary shapes with a well-defined porous interior will be accessible. Namely, the 3D printing technique in combination of a matching post-synthetic processing will offer an innovative technological platform for producing catalytic electrodes, especially in terms of customizable structural features and morphologies^10,11^.

Among preparative protocols of traditional catalytic electrodes, the electrocatalyst powder-based process is fairly common. It usually needs to be shape-fixed by a polymeric binder, e.g., nafion or polyvinylidene fluoride, to glue the powderous catalysts onto carbon cloths or glassy carbon carriers^12,13^. An alternative is the self-supporting form, normally accessed by an in situ growth method^14,15^. Generally, this method may require a long reaction time and/or a high-temperature/-pressure treatment. The structure of self-supporting electrodes is mainly dependent on the chosen substrate and the deposition conditions, and the accurate control of their shape and fine structures can be challenging. In this regard, the 3D printing technique when paired with a suitable post-printing processing opens up a vastly unexplored dimension^16^. Depending on the application scenario, task-specific printing inks can be individually formulated and employed as gel-like precursors to create the electrode structures via 3D printing^17^. That is to say, direct transformation from a printing ink to the desirable self-supporting carbon electrode will be realized regardless of additional carriers, such as carbon black or graphene^18^. Unlike traditional methods, the 3D printing technique can more precisely shape the micron-level architecture of electrodes by designing and adjusting the printing parameters^19,20^. It is expected to provide an excellent technological option for the design and creation of customized, self-supporting electrodes for target electrocatalysis.

In this study, self-supporting metal-free porous carbon electrodes with B, P, N-ternary doping, termed BPN-3Dp-CCEs, were made by 3D-printing ionic liquid with the assistance of the follow-up conformal carbonization. The as-obtained BPN-3Dp-CCEs were investigated, structurally optimized and used in electrocatalytic CO_2_ reduction reaction (CO_2_RR) to produce syngas with tailorable composition. This approach points out an alternative choice for engineering advanced multi-heteroatoms-doped porous carbon electrodes for catalysis in and beyond CO_2_RR.

## Results and discussion

**Fig. 1:**
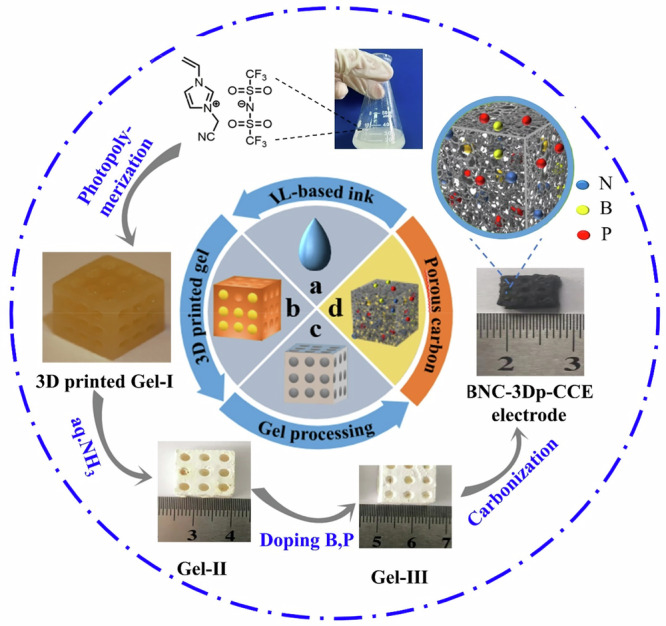
Synthetic scheme of B, P, N-ternary doped porous carbon electrode BPN-3Dp-CCE from an ionic liquid-based ink. The chemical structure of the ionic liquid is shown on the upper-left.**a** The monomeric ionic liquid-based ink; **b** 3D-printed Gel-I (before any post-printing processing); **c** 3D-printed Gel-II (after immersion in aqueous NH_3_); **c** 3D-printed Gel-III (after doping with tetraphenylphosphonium tetraphenylborate; **d** The as-obtained BPN-3Dp-CCE electrode. The photograph shows the dimension of the electrode. Its enlarged view is a cartoon highlighting the heteroatoms in the carbon matrix.

Figure 1 is a schematic of the preparation procedure of carbon electrodes in three steps, i.e., the preparation of an ionic liquid-based printing ink, the 3D printing process to form the gel and its follow-up modification, and the carbonization as the final step. The final carbon product BPN-3Dp-CCE serves as a catalytic electrode.

In detail, the ink preparation began with the dissolution of a chosen ionic liquid monomer 3-cyanomethyl-1-vinylimidazolium TFSI (termed CMVImTFSI) in DMSO (Fig. 1a). To this solution, PAA, a diacrylate crosslinker, and a photoinitiator were added as additives and fully dissolved to form the target ionic liquid-based 3D-printing ink. To note, each component in the ink plays an inevitable role. Concretely, the CMVIm^+^ cation in the ionic liquid monomer CMVImTFSI is the major source of carbon and nitrogen, where the TFSI^−^ anion can volatilize at *ca*. 400 ^o^C during the pyrolysis process to leave micropores behind in the carbon matrix. The diluent DMSO is used to regulate the viscosity of the printing ink, so to match the photo curing rate to cure the freshly printed ink layer. PAA, as an ionic crosslinker upon NH_3_ treatment, is used to enhance the mechanical stability of the printed gel, and to produce pores in the gel that can be preserved in the final carbon electrode^21^. Last, the divalent crosslinking agent strengthens the printed precursor’s framework to render the mechanical stability. All these components together constitute a 3D-printing mixture ink with the ionic liquid monomer as the main component.

In Fig. 1b, the **Gel-I** was pre-designed by CAD software and printed out from the mixture ink by a 3D printer. It appeared light orange in color and had a number of cylindrical channels distributed across **Gel-I** to increase its surface area. After 3D printing, the **Gel-I** was immersed in an aqueous NH_3_ solution. The NH_3_ solution acted as an etchant for solvent exchange with DMSO in **Gel-I**. Finally, water was removed by a subsequent freeze-drying process to form **Gel-II** (Fig. 1c). To note, during the immersion step in aqueous NH_3_ solution, the NH_3_ molecules reacted with the carboxylic acid group (-COOH) of the PAA chains to neutralize PAA into its polyanion form poly(ammonium acrylate) that immediately ionically complexed with the in situ formed poly(ionic liquid) formed by photopolymerization in the 3D printing step. In addition, the diffusion of aqueous NH_3_ solution into the **Gel I** led to the structural rearrangement and pore formation, a mechanism similar to our previous report to make a porous polymer membrane^22^. These pores will be replicated in the final carbon electrode to expose more surface for heterogeneous reactions^23^.

This study focuses mainly on the preparation of 3D-printed carbon-based catalytic electrodes from heteroatom-rich ionic liquid. After carbonization, the electrodes were first screened with catalytic properties in the CO_2_RR application. It was reported previously that doping N atoms into carbon materials could alter their bulk electronic structure and surface properties, which are conducive to the formation of active centers involved in the electrocatalytic reactions^24,25^. Meanwhile, B and P atoms are also considered as suitable heteroatomic dopants to carbons^26,27^. Here the 3 types of heteroatoms B, P, and N are coupled together to promote charge redistribution in the catalytic electrode materials^28,29^. To introduce the B and P species, **Gel-II** (Fig. 1c) was immersed in a solution of tetraphenylphosphonium tetraphenylborate at various concentrations. This step allowed the B and P species to penetrate into the **Gel-II** to form the final carbon precursor **Gel-III** that possessed N, P and B species. Last, **Gel-III** was carbonized at pre-selected temperature to produce the self-supporting B, P, N-ternary doped carbon electrodes BPN-3Dp-CCEs, as shown in Fig. 1d. Notably, it shrank ~50% in dimention after carbonization, while remaining its microstructures and shape.

The as-made carbon electrodes were initially screened in the CO_2_RR tests in a H-type electrolytic cell (Supplementary Fig. S2). In the CO_2_RR process, the porous structure and the ternary elemental dopants of B, P, and N provide a good basis for the efficient mass transfer process and catalytically active sites, respectively, on the BPN-3Dp-CCE^30^. As a control experiment, *via* the same preparation process, **Gel-II** without treatment by tetraphenylphosphonium tetraphenylborate (thus without B, P dopants) was carbonized into a B,P-free control sample termed N-3Dp-CCE. In the electrochemical performance study, the optimal BPN-3Dp-CCE catalytic electrode was obtained by studying the impact of the carbonization temperatures and the treatment of **Gel II** at different concentrations of tetraphenylphosphonium tetraphenylborate. Their specific performance in the CO_2_RR process was evaluated and the results are shown in supporting information of Supplementary Fig. S3 and Fig. S4. It was found that too high or too low pyrolysis temperatures were adverse to CO_2_RR of the carbon electrode. It is worth noting that a higher pyrolysis temperature favors a higher graphitization degree and thus better conductivity, but lowers down the concentration of heteroatoms in carbons that are the active sites for catalysis^31^. Due to this trade-off, only at an appropriate pyrolysis temperature, the optimal sample BPN-3Dp-CCE for CO_2_RR can be obtained^32^. Meanwhile, the amount of B, P heteroatoms introduced into the BPN-3Dp-CCEs also plays an enabling role on the catalytic activity. The experiment results in supporting information of Supplementary Fig. S3 and Fig. S4 show that the BPN-3Dp-CCE prepared by B,P-doping treatment at 60 g L^−1^ and a final carbonization temperature at 1050 °C shows the optimal FE_CO_ and current density, thus was chosen to be studied further.

**Fig. 2:**
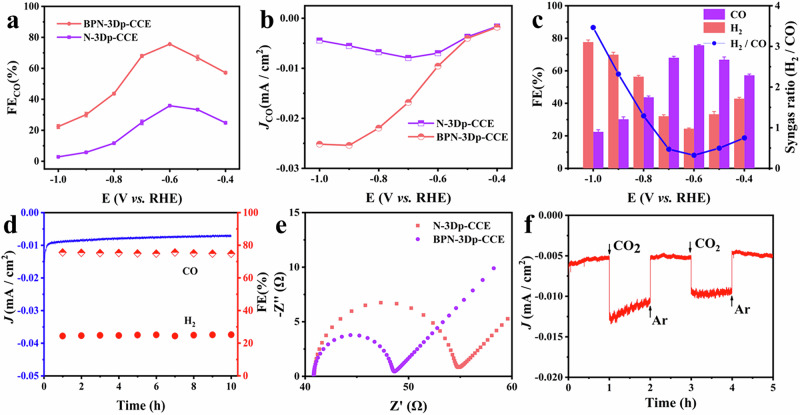
Electrochemical performance tests of BPN-3Dp-CCEs and the reference N-3Dp-CCE that contains N as the heteroatom. **a** Plot of Faradaic efficiency of CO (FE_CO_); **b** Plot of the partial current densities of CO (*j*_CO_); **c** Plot of overall Faradaic efficiency (left) and the syngas ratio (right) on BPN-3Dp-CCE at different potentials; **d** Stability test of BPN-3Dp-CCE catalytic electrode at −0.6 V; **e** Nyquist plots of BPN-3Dp-CCE and N-3Dp-CCE; **f** Current-time plots of Ar and CO_2_ by alternating the inlet at −0.6 V on BPN-3Dp-CCE.

Figure 2a shows the FE_CO_ of the optimal BPN-3Dp-CCE and its reference N-3Dp-CCE electrode at various potentials. Both samples present the same trend of gradual rise in FE_CO_ from −0.4 V to −0.6 V, peaking at −0.6 V, and then stepwise dropped to the lowest value at −1.0 V (Comprehensive data are presented in Supplementary Table S1). Clearly, the optimal BPN-3Dp-CCE exhibits a higher FE_CO_ at tested potentials than that of N-3Dp-CCE, highlighting the significance of the heteroatoms in promoting the CO_2_RR.

As the hydrogen evolution reaction is a competing reaction, the FE_H2_ was studied and shows a completely opposite trend to the FE_CO_ data observed on the same electrode (Supplementary Fig. S5a). Compared with the N-3Dp-CCE, the BPN-3Dp-CCE shows an advantageously lower FE_H2_ at all potentials. Doping with multiple heteroatoms may induce, beside more active sites, synergistic effects. This phenomenon is verified in this study by B, P, N-ternary doping simultaneously in the BPN-3Dp-CCE for CO_2_RR application. It is generally believed that N doping enhances the catalytic performance by improving the conductivity, surface wettability and chemical affinity of the electrode^33,34^. Meanwhile, P has a large atomic size, valence bonding and good electron-donating ability^35^ and B dopant can regulate the local charge density and surface charge state^36^. All of these effects jointly promote the CO_2_RR performance of BPN-3Dp-CCE.

The local current density of CO (*j*_co_) on the BPN-3Dp-CCE and N-3Dp-CCE were compared. As shown in Fig. 2b, as the reduction potential increases, the *j*_co_ first increases till a maximum, and then reduced. At −1.0 V, the *j*_co_ of the BPN-3Dp-CCE is 0.0252 mA cm^−2^, while that of the N-3Dp-CCE is only 0.0040 mA cm^−2^. Obviously, the BPN-3Dp-CCE has a much larger *j*_co_. As such, when the applied potential is low, the active sites favorable for CO production on the catalytic electrode are not fully activated, thus showing a lower CO productivity. Next, as the applied potential increases gradually, more active sites are activated and thus *j*_co_ increases accordingly. However, there is a fixed number of active sites in the sample. When all active sites are activated, the catalytic CO_2_ reduction for production of CO reaches the maximum. Thus, as the applied potential further rises, there is no significant growth on *j*_co_. At the same time, the local current density for H_2_ (*j*_H2_) on the BPN-3Dp-CCE is favorably close to 0 (Supplementary Fig. S5b).

To compare the Faradaic efficiency, the FE_CO_ and FE_H2_ of the BPN-3Dp-CCE are shown in Fig. 2c, with the highest FE_CO_ (75.6%) and the lowest FE_H2_ (24.4%) being reached at −0.6 V. At the same time, the tunable composition of syngas at different potentials is studied, with the lowest H_2_:CO molar ratio of 0.32 at −0.6 V, and the highest of 3.46 at −1.0 V. Clearly, by altering the applied potential, compared with other catalysts for syngas production (Supplementary Table S2), the ratio (H_2_:CO) of the syngas made on the BPN-3Dp-CCE can be adjusted between 0.32 and 3.46. It is considered an advantage that the syngas produced by the BPN-3Dp-CCE covers a wide composition range so to meet different industrial requirements^37^.

Serving as a catalytic electrode, stable performance is important. Figure 2d presents the stability test of the BPN-3Dp-CCE for 10 h. It can be seen that the FE_CO_ is kept above 70% and FE_H2_ below 30% after electrolysis at −0.6 V. Compared with the initial point, Faradaic efficiency remains practically unchanged. In addition, the decay in current density is neglectable after 10 h, indicating long-term stability of the BPN-3Dp-CCE in catalytic operation.

To investigate the effect of extra dopants of B and P elements beside the N, on the performance of CO_2_RR, the Nyquist diagrams of N-3Dp-CCE and BPN-3Dp-CCE are displayed in Fig. 2e. It is clearly seen that the BPN-3Dp-CCE has a lower interfacial charge transfer resistance, which can be attributed to the fact that the ternary dopants in carbon skeleton with B, P, and N can produce a synergistic coupling effect^30^. It has been previous reported that the synergistic effect of B, P, and N atoms can better promote charge transfer, optimize charge re-distribution, and improve electrical conductivity of the original carbon material^29^.

Finally, since it needs to be evident about the source of CO_2_ in the CO_2_RR process, Fig. 2f shows a controlled trial in both Ar- and CO_2_-saturated electrolytes. The current density of the BPN-3Dp-CCE in CO_2_-saturated electrolyte is greater than that in Ar-saturated electrolyte. Through this phenomenon, it can be proven that it is the CO_2_ dissolved in the electrolyte that runs the CO_2_RR, not the CO_2_ entering the reactor before CO_2_RR. To further verify this observation, the FE of reduction products was determined in Ar- or CO_2_-saturated electrolyte on BPN-3Dp-CCE, and the response curves of the thermal conductivity detector (TCD) and flame ionization detector (FID) of the gas chromatography further confirm this result (Supplementary Fig. S6).

**Fig. 3:**
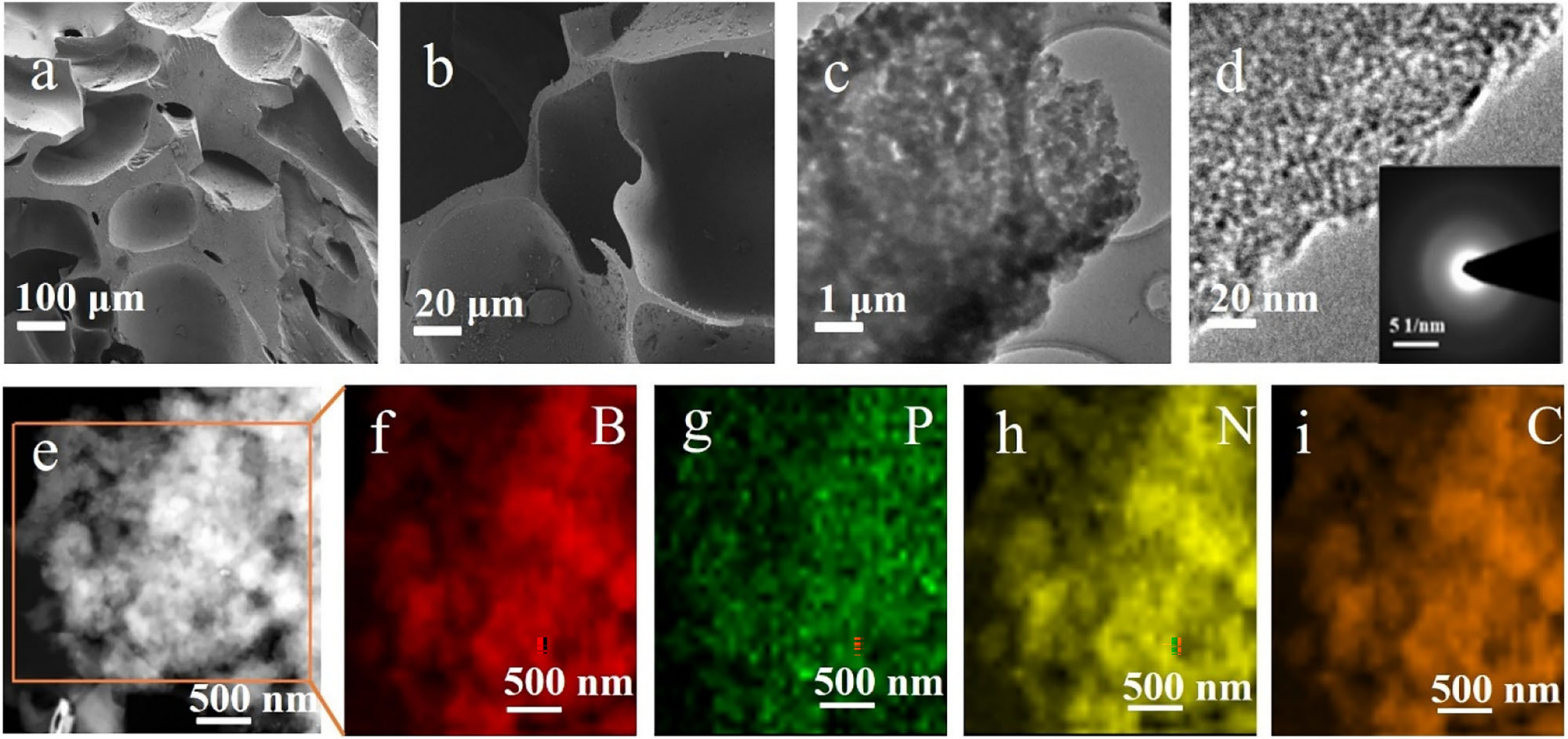
Structural and elemental characterization of BPN-3Dp-CCE. ** a, b** Representative SEM images of the BPN-3Dp-CCE; **c, d** Representative TEM images of BPN-3Dp-CCE, the inset in (**d**) corresponds to its selected area electron diffraction (SAED) image; **e** HAADF-STEM image of BPN-3Dp-CCE; Elemental mapping images of B (**f**), P (**g**), N (**h**), and C (**i**) in BPN-3Dp-CCE.

To better understand the morphological and structural characteristics of BPN-3Dp-CCE, Fig. 3a, b shows its scanning electron microscope (SEM) images. Clearly, there are large pores distributed in the BPN-3Dp-CCE. The reason for the formation of these large pores in Fig. 3a can be attributed to the solvent residue trapped in the print body. Its volatilization during the pyrolysis process results in the pore structure. Figure 3b is an enlarged view of the pores showing an inner rough surface. Figure 3c, d are transmission electron microscopy (TEM) images of the BPN-3Dp-CCE. It can be seen that the BPN-3Dp-CCE has a distinct porous structure. The formation of micropores is due to the fact that the TFSI^−^ anion in the ionic liquid species volatilized during pyrolysis of the **Gel-III**, leaving behind micropores in the final BPN-3Dp-CCE. The macropores are formed by immersing the printed body into ammonia to allow solvent exchange, while the ammonia reacts sufficiently with PAA in the printed **Gel-I**^38,39^. The mesopores are supposed to be built up by the fragmentation of polymers in the **Gel-III** along the pyrolytic degradation. All pores jointly enable a large accessible surface area, offering rich active sites and enhanced charge transfer, thereby facilitating good contact between the CO_2_ and the electrolyte. Furthermore, the N₂ adsorption-desorption isotherms indicate the coexistence of micropores, mesopores, and macropores (Supplementary Fig. S7). The micropores increases the specific surface area, facilitating charge accumulation, while the mesopores and macropores enhance kinetics in gas diffusion and transport, thereby raising CO_2_ conversion efficiency. The inset of Fig. 3d is a selected area electron diffraction (SAED) pattern, which proves the polycrystalline nature of the BPN-3Dp-CCE. Figure 3e shows the high angle annular dark field scanning transmission electron microscope (HAADF-STEM) image of the optimal BPN-3Dp-CCE. As seen in Fig. 3f–i, the B, P, and N elements have been successfully doped in carbons and distributed uniformly in the whole region.

**Fig. 4:**
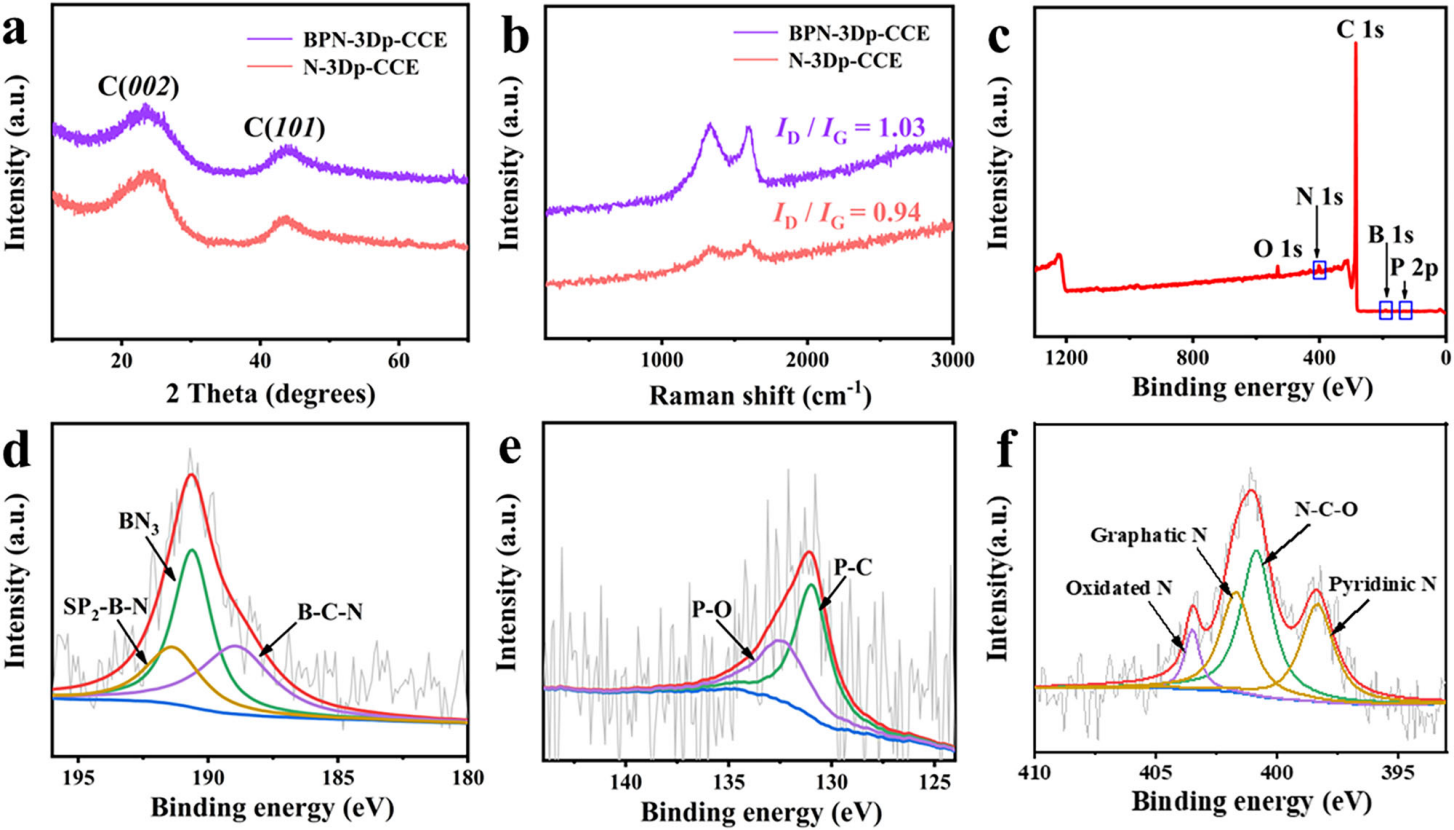
Structural and surface chemical analysis of BPN-3Dp-CCE and N-3Dp-CCE. XRD patterns (**a**) and Raman spectra (**b**) of BPN-3Dp-CCE and N-3Dp-CCE; The full XPS spectrum of BPN-3Dp-CCE (**c**); The high-resolution XPS spectra of B 1s (**d**), P 1s (**e**) and N 1s (**f**).

X-ray diffraction (XRD) is applied here to study the phase structure. The XRD patterns of BPN-3Dp-CCE and N-3Dp-CCE were recorded in Fig. 4a. It can be seen in both diagrams that there are two broad diffraction peaks at 23.6° and 44.1°, corresponding to the reflections of (*002*) and (*101*) crystal planes of carbons, respectively. Compared with N-3Dp-CCE, the characteristic XRD peaks of the BPN-3Dp-CCE show no obvious shift in comparison to N-3Dp-CCE, which indicates that mono- and ternary-doping by heteroatoms have similar effects on the formation of graphitic phase. Figure 4b is the Raman spectra of the BPN-3Dp-CCE and N-3Dp-CCE. The *I*_D_/*I*_G_ ratio is used often to evaluate the density of defects in carbon materials^40^. The *I*_D_/*I*_G_ values of BPN-3Dp-CCE and N-3Dp-CCE are measured to be 1.03 and 0.94, respectively. It indicates that the BPN-3Dp-CCE has more structural defects and thus carries more active sites, which is consistent with the electrochemical results studied above.

To investigate the elemental composition and valence state of the BPN-3Dp-CCE, it was further subject to XPS analysis. Figure 4c is the XPS full spectrum of the BPN-3Dp-CCE, indicating the presence of B, P, N, O, and C elements. The mass percentage of elements B, P, N, and C was quantified to be 0.89%, 2.15%, 0.09%, and 95.12%, respectively, confirming the incorporation of B, P, and N into the carbonaceous catalytic electrode. Figure 4d–f are the high resolution XPS spectra of B 1s, P 2p, and N 1s for the BPN-3Dp-CCE electrode, respectively. As shown in Fig. 4d, there are three fitted peaks at 188.6, 190.4 and 191.5 eV in the high-resolution XPS spectrum of B 1s, corresponding to B-C-N, BN_3_ and sp^2^-B-N bonds, respectively^41^. In Fig. 4e, there are two fitted peaks corresponding to P-C and P-O bonds in the high-resolution XPS spectrum of P 2p at 132.1 eV and 133.2 eV, respectively^42^. Figure 4f shows the high-resolution XPS spectrum of N 1s on the BPN-3Dp-CCE. There are four fitting peaks assigned for four kinds of nitrogen species, i.e., pyridinic N (398.4 eV), N-C-O (400.7 eV), graphitic N (402.5 eV), and oxidic N (406.4 eV)^43,44^. Specifically, B dopant in BPN-3Dp-CCE can increase the in-plane defects of the material^36^, while P has a lower electronegativity and higher electron-donating capability^35^, which can provide additional active sites in BPN-3Dp-CCE. Meanwhile, pyridinic N and pyrrolic N help improve the electrochemical performance of BPN-3Dp-CCE for CO_2_ reduction. In addition, graphitic N and oxidic N better the electrical conductivity and electron transfer of BPN-3Dp-CCE. Importantly, the synergistic interaction among these three heteroatoms has been reported previously to break the electroneutrality of C and optimizes the charge distribution of the pristine carbon material, thus promoting charge transfer and catalytic activity^45^.

According to the above-mentioned results and the comparison with other porous carbon-based catalysts (Supplementary Table S3), the self-supporting carbon catalytic electrode fabricated via 3D printing demonstrates high promising prospects for future applications.Above all, the 3D-printed electrodes are obtained by stacking the 2D planes over each other layer-by-layer and the following pyrolysis process of the gel precursor. As a result, 3D printing can transform the 3D solid into individual 2D planes, which ensures the good controllability of its preparation. In addition, compared with conventional powder-based electrodes, the 3D-printed electrodes have intuitive structural features and good mechanical stability. Meanwhile, the molding process of 3D printing electrodes is simple, and the gel precursors for electrode production can be manufactured in large quantities according to the specified parameters. As 3D printing technology has been developed more maturely nowadays, the industrial production and application of the 3D printing technique in catalytic carbon electrodes show great potential.

## Conclusions

To sum up, self-supporting porous carbon-based catalytic electrodes with B, P, N-ternary doping for CO_2_RR were prepared by combining the 3D printing technique with the conformal carbonization. When the as-obtained BPN-3Dp-CCE electrode was used to reduce CO_2_ into syngas, the FE_CO_ and FE_H2_ reach up to 75.6% and 24.4% at the potential of −0.6 V, respectively. By adjusting the reduction potential, the H_2_:CO ratio of syngas can be adjusted broadly from 0.32 to 3.46 to satisfy a wide range of use. Notably, after 10 h of continuous electrolysis, the Faradaic efficiency and current density remain basically unchanged. This work provides an innovative approach for the future design of self-supporting catalytic carbon electrodes with a high level of control in structures.

## Methods

### Materials

Diphenylphosphine oxide (97%), 1,4-bis(acryloyloxy)butane (analytic grade), and poly (acrylic acid) (PAA) (analytic grade) were purchased from Aladdin Reagent Factory. Tetraphenylphosphonium tetraphenylborate (C_48_H_40_BP) (biotechnology grade) was obtained from Shanghai Jingchun Reagent Co., Ltd. Dimethyl sulfoxide (DMSO) ( ≥ 99.5%) and aqueous ammonia solution (analytic grade) were sourced from Sinopharm Chemical Reagent Co., Ltd. Potassium bicarbonate (KHCO_3_) (analytic grade) was acquired from Saen Chemical Technology (Shanghai) Co., Ltd. Bis(trifluoromethanesulfonyl)imide lithium salt (LiTFSI, 99%) was purchased from Macklin. All reagents were used as received without further purification. The used ionic liquid monomer 3-cyanomethyl-1-vinylimidazolium TFSI was homemade and its chemical structure was shown in the up-left corner of Fig. 1 and Supplementary Fig. S1.

### Preparation of carbon electrodes

The preparation of catalytic electrodes was completed in the following three steps.

#### Preparation of the 3D printing ink

In a typical run, 1.00 g of the chosen ionic liquid monomer (chemical structure shown in the up-left corner of Figs. 1 and S1) was added into 4.00 g of DMSO. The mixture was stirred into a homogeneous solution. Then, 1.60 g of PAA (***M***_***W***_ of 250 kDa) was dissolved in 3.00 g of DMSO and mixed with the above solution. Afterwards, 1.28 g of the crosslinker 1,4-bis(acryloyloxy)butane and 0.70 g of photoinitiator diphenylphosphine oxide were added. The 3D printing ink, which was suitable for light-curing 3D printers, was obtained after stirring for 2 h.

#### 3D printing of the gel precursor

The CAD software was used to create a digital model of the print body in a size of 1.5 x 1.5 x 0.5 cm. A number of holes in a diameter of 0.22 ± 0.02 cm were engineered through the model to increase the surface area of the electrodes and to improve the mass transfer efficiency. The model was then converted to a STL format and sliced using a photon software into a large number of 2D cross-sectional layers. Finally, the light-curing 3D printer deposited the 2D cross-sectional layers of the material layer-by-layer until the desired model was obtained. During the printing process, the exposure time of the model was set to 16 s and the corresponding slice thickness was 0.05 mm.

#### Preparation of self-supporting carbon electrodes

The afore-mentioned 3D-printed fresh gel (termed **Gel I**) was immersed in a 0.25 wt% 5 mL aqueous NH_3_ solution for 48 h, where the ammonia solution was refreshed every 6 h. The treated gel was then freeze-dried (termed **Gel II**). Afterwards, the **Gel II** was further treated by a tetraphenylphosphonium tetraphenylborate (C_48_H_40_BP) solution in DMSO at different concentrations of 0, 20, 40, 60, and 80 g L^−1^. Annealing treatment at 100 °C for 10 h in an autoclave was conducted to promote the boron and phosphorus sources to penetrate into the gel to form the final gel precursor (termed **Gel III**). Finally, the 3D printed carbon-based catalytic electrodes were obtained by carbonization of **Gel III** at various temperatures (950 °C–1100 °C) under Ar gas protection. The carbonization treatment was first ramped up to 300 °C at a rate of 3 °C min^−1^ and held at this temperature for 1 h, followed by heating to the final temperature of 950, 1000, 1050, or 1100 °C and held at the final temperature for 1 h. Lastly, the oven was allowed to cool down to room temperature in 5 h.

### Measurements and characterizations

To test the performance of the as-prepared 3D-printed carbon electrode for CO_2_RR, a three-electrode system with a H-shaped electrolytic cell was used on a CHI 660E electrochemical workstation. The working electrode is the as-prepared 3D-printed carbon electrode, the reference electrode uses a calomel electrode, and the counter electrode is a platinum mesh. In the cathode chamber where the working electrode was located, high-purity CO_2_ was introduced at a flow rate of 24 mL min^−1^ for 30 min to allow sufficient CO_2_ gas to enter and saturate the solution. Then it was electrochemically converted. All electrochemical tests used 0.1 M KHCO_3_ solution as electrolyte at room temperature.

Physical characterizations of BPN-3Dp-CCEs were conducted as follows. Scanning electron microscopy (SEM) was recorded on a ZEISS Gemini SEM 550. The acceleration voltage was adjusted in the range of 0.02–30 kV, with a theoretical resolution of 0.5 nm at 15 kV. Transmission electron microscopy (TEM) and energy dispersive X-ray spectroscopy (EDX) were recorded on a FEI TECNAI G2 TF20 S-TWIN TMP microscope (America). X-ray power diffraction (XRD) measurements were carried out on a Rigaku 92 D/Max-2400 diffractometer (Japan) employing Cu K_α_ radiation at 40 kV and 150 mA (scan rate: 10 °min^−1^). Raman spectra were recorded on a Bruker RFS100/S spectrometer (Germany; laser wavelength: 663.8 nm). X-ray photoelectron spectroscopy (XPS) data were obtained on a Kratos Axis Ultra DLD spectrometer (Japan) using radiation source Al K_α_ at an energy of 1486.6 eV.

## Supplementary information

Supplementary Information

## Data Availability

The data that support the findings of this study are available from the correspondence author upon reasonable request.
