# Supplementary material for: Multi-heteroatom-doped porous carbon electrodes from 3D printing and conformal carbonization of ionic liquids for electrocatalytic CO2 conversion into syngas
Source: Commun Chem. 2025 Apr 23;8:121. doi: 10.1038/s42004-025-01514-1 (PMC12019238; doi:10.1038/s42004-025-01514-1)
Supplement: Supplementary file 1 — Supplementary Information [file 42004_2025_1514_MOESM1_ESM.pdf]

Supplementary Information

Multi-heteroatom-doped porous carbon electrodes from 3D printing and conformal carbonization of ionic liquids for electrocatalytic CO_2 conversion into syngas

Wei Wang[1, *], Na Zhao[1], Kai Zhao[1], Miao Zhang[2], Kanglei Pang[2], Yu Zhang[2], Jiayin Yuan[2, *]

[1] *School of Chemistry and Chemical Engineering, Lanzhou Jiaotong University, Lanzhou 730070, China*

[2] *Department of Materials and Environmental Chemistry (MMK), Stockholm University, Stockholm 10691, Sweden*

1. Related Experimental Information

On-line gas chromatograph (GC: FL9790II, the chromatographic column is equipped with a 5A molecular sieve. Hayesep Q and PorapLOT Q) was used to analyze the gas products after electrolysis. The carrier gas is nitrogen (N_2). A thermal conductivity detector (TCD) is used to detect H_2 at 120 °C for a retention time of about 4 min. The flame ionization detector (FID) is used to detect carbon monoxide

[*] Corresponding author, e-mail: wangw@lzjtu.edu.cn (W. Wang)； jiayin.yuan@mmk.su.se (JY. Yuan).

and other hydrocarbons at 200 °C with a retention time of about 11.5 min. Calibration mixtures with the following compositions were used: 20.3 ppm C_2H_2, 503.3 ppm CO, 504.2 ppm H_2, and 51.6 ppm CH_4; 101.5 ppm C_2H_2, 2469.8 ppm CO, 2529.8 ppm H_2, and 250.8 ppm CH_4; 205.5 ppm C_2H_2, 5015.8 ppm CO, 5019.7 ppm H_2, and 501.2 ppm CH_4, supplied by Dalian Date Gas Co., Ltd. External standard curves were drawn with three kinds of standard gas mixtures with different proportions, and the experimental gases were measured by external standard method.

The CO_2 flow rate was set to 24 mL min^{-1} and the chromatogram was injected 1 mL each time. A partial current of the gas phase product can be calculated based on the concentration of the test gas, divided by the apparent total current, which is the faraday efficiency of the corresponding product. The partial current of the product is calculated as follows:

$$I_0(\mathrm{i}) = \mathrm{x_i n_i F} v_\mathrm{m}$$

where x_i is the gas content measured by on-line gas chromatography, and n_i is the number of reduced electrons corresponding to different products. F is the Faraday constant (96485 C / mol). v_m is the molar CO_2 gas flow rate (24 mL min^{-1} / 24.5 mol L^{-1}). The corresponding Faradaic efficiency at each potential is calculated as follows:

$$\mathrm{FE} = \mathrm{I_0} / \mathrm{I_{total}} \times 100\%$$

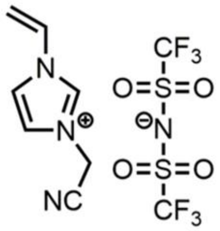

Fig. S1 The chemical structure of the chosen ionic liquid monomer.

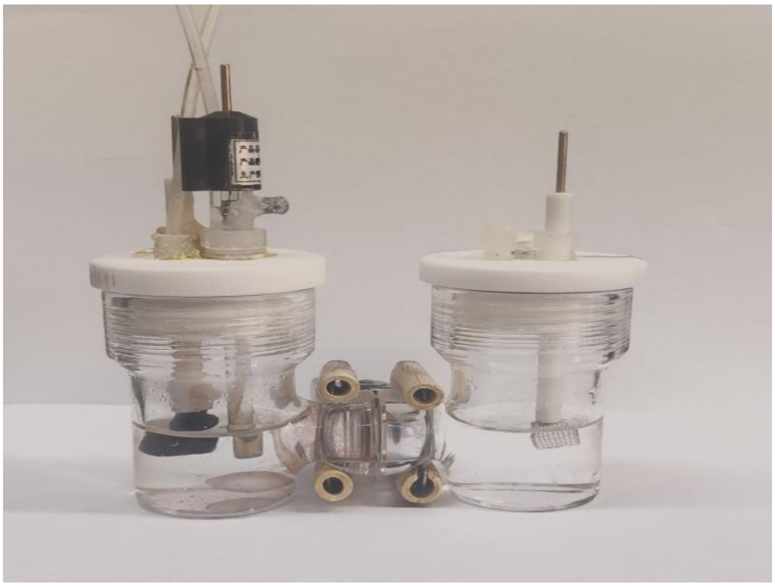

Fig. S2 Photograph of the H-type electrolytic cell for CO_2RR test. The electrolyte is 0.1 M aq. $KHCO_3$ solution.

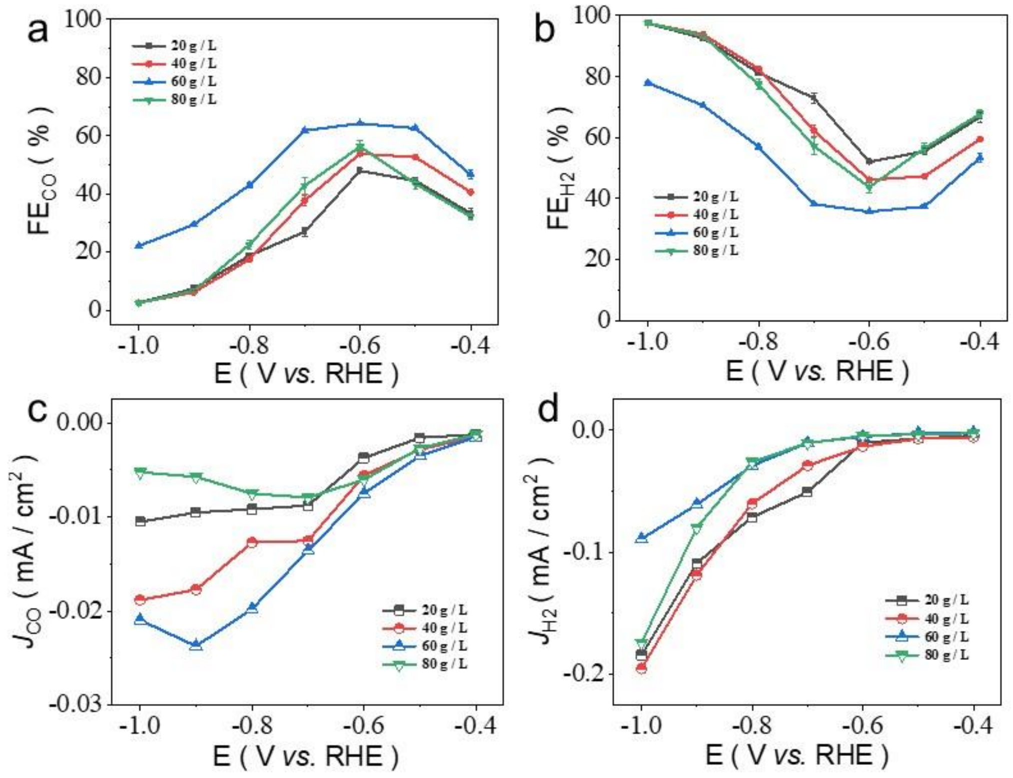

Fig. S3 FE$_{CO}$ **(a)** and FE$_{H2}$ **(b)** of CO_2RR on BPN-3Dp-CCE electrode prepared by treating the **Gel II** at different concentrations of aqueous tetraphenylphosphonium tetraphenylborate ($C_{48}H_{40}BP$) solution before carbonization; j_{CO} **(c)** and j_{H2} **(d)** of CO_2RR on BPN-3Dp-CCE electrode prepared by treating the **Gel II** at different concentrations of aqueous tetraphenylphosphonium tetraphenylborate ($C_{48}H_{40}BP$) solution before carbonization.

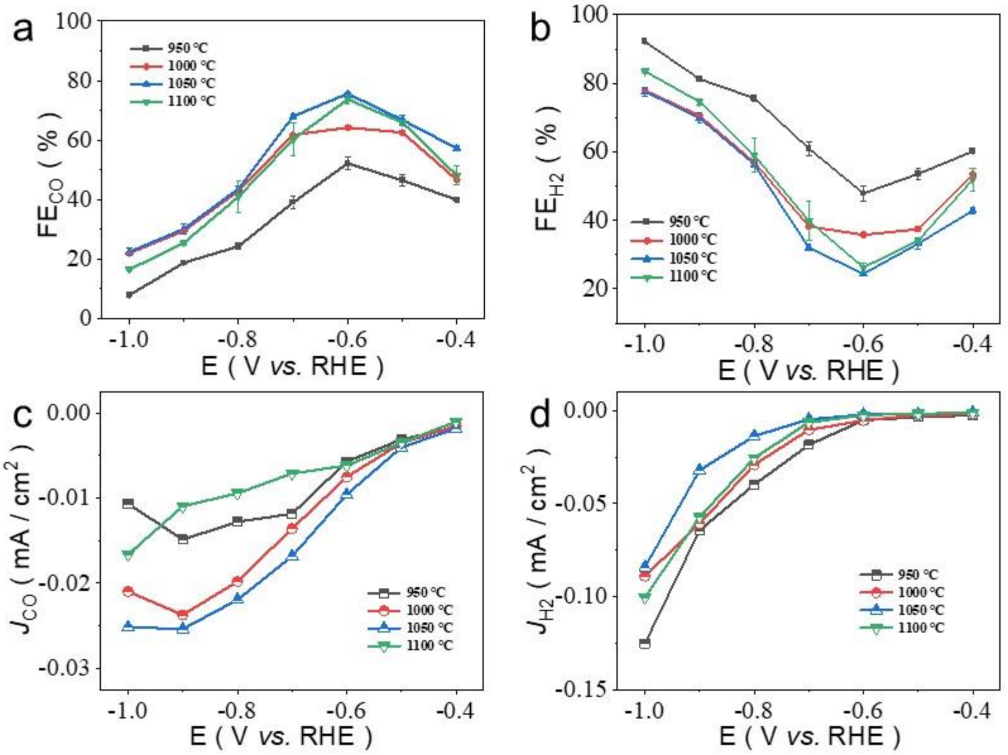

Fig. S4 FE_{CO} **(a)** and FE_{H2} **(b)** of CO_2RR on BPN-3Dp-CCE electrode prepared at different final temperatures; j_{CO} **(c)** and j_{H2} **(d)** of CO_2RR on BPN-3Dp-CCE electrode prepared at different final temperatures.

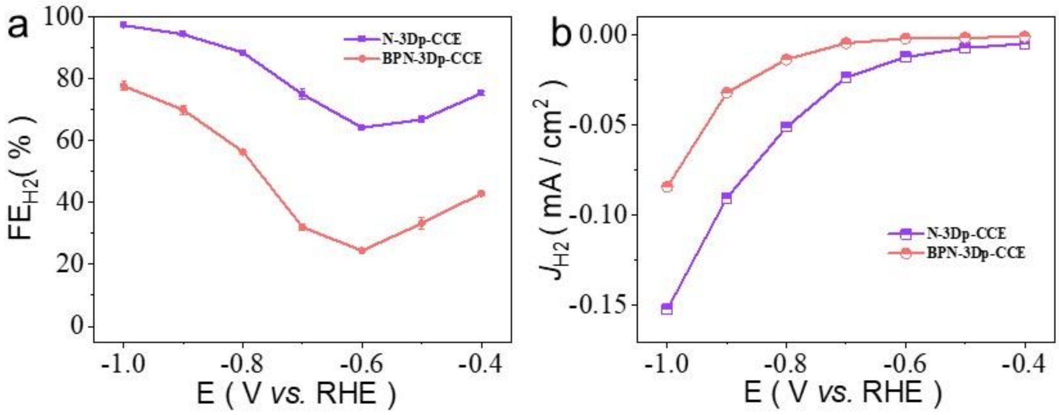

Fig. S5 Electrochemical performances of BPN-3Dp-CCE and N-3Dp-CCE electrodes on Faradaic efficiency of H_2 (FE_{H2}) **(a)**, and the partial current densities of H_2(j_{H2}) **(b)**.

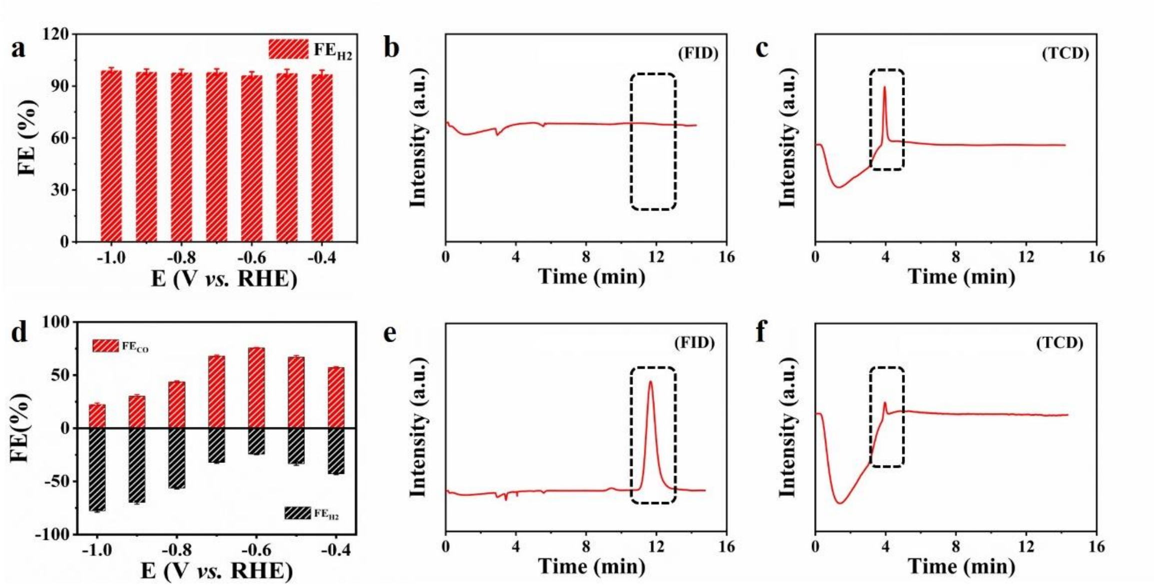

Fig. S6 FE histogram of BPN-3Dp-CCE under Ar-saturation **(a)** and gas chromatograms on the corresponding FID detector **(b)** and TCD detector **(c)**; FE histogram of BPN-3Dp-CCE under CO_2-saturation **(d)** and gas chromatograms on the corresponding FID detector **(e)** and TCD detector **(f)**.

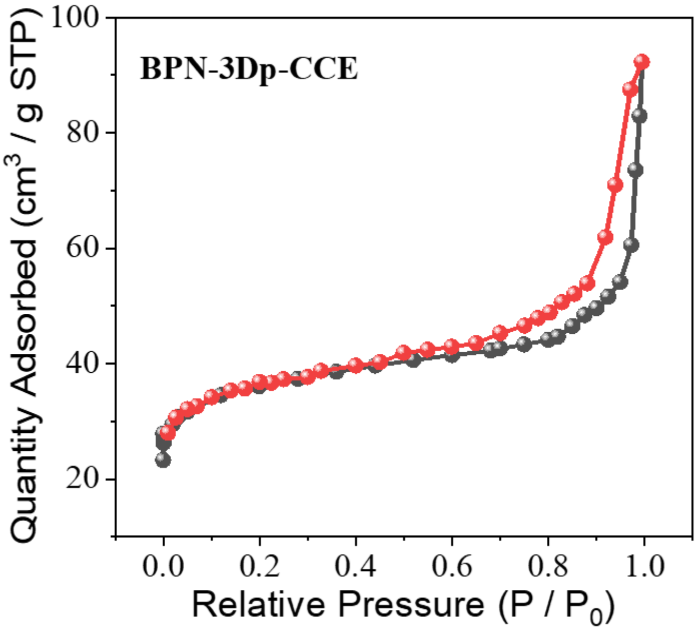

Fig. S7 The nitrogen adsorption-desorption isotherms of the optimal sample BPN-3Dp-CCE.

Table S1. The comparison of FE_{CO} at different potentials for the optimal BPN-3D-CCE and N-3D-CCE.

**Potential (V)**	**FE_{CO} of BPN-3Dp-CCE (%)**	**FE_{CO} of N-3Dp-CCE (%)**
**-0.4**	57.2	24.8
**-0.5**	66.8	33.3
**-0.6**	75.6	35.9
**-0.7**	68.0	25.1
**-0.8**	43.7	11.7
**-0.9**	30.1	5.8
**-1.0**	22.4	2.8

Table S2. Comparison of BPN-3Dp-CCE with other electrocatalysts in literature for syngas production with different H_2:CO ratios.

Catalysts	Potential(V)	Ratio of H_2:CO	Electrolytes	Ref
**BPN-3Dp-CCE**	**-0.40~-1.00 (*vs.* RHE)**	**0.32~3.46**	**0.1 M $KHCO_3$**	**This work**
**BiZn/NC**	-0.36~-0.8 (*vs.* RHE)	0.20~2.92	0.5 M $KHCO_3$	[1]
**Br-Ag (OR)**	-0.50~-1.10 (*vs.* RHE)	0.33~0.50	saturated NH_4HCO_3	[2]
**Ag/TiO_2**	-0.30~-0.70 (*vs.* RHE)	0.10~1.50	1 M KOH	[3]
**R-Ag/TiO_2/p-Si**	-0.20~-0.90 (*vs.* RHE)	0.33~1.00	0.1 M $KHCO_3$	[4]
**CoNC-400**	-2.00~-2.60 (*vs.* Fc/Fc^+)	0.94~1.18	acetonitrile solution	[5]
**CdS**	-0.70~-1.20 (*vs.* RHE)	0.25~2.30	0.1 M $KHCO_3$	[6]
**V_O-rich ZnO**	-0.70~-1.10 (*vs.* RHE)	0.20~0.60	0.1 M $KHCO_3$	[7]
**Co@CoNC-900**	-0.50~-0.80 (*vs.* RHE)	0.50~1.50	0.1 M $KHCO_3$	[8]
**NC (NH_3)**	-0.50~-0.80 (*vs.* RHE)	0.20~0.67	0.1 M $NaHCO_3$	[9]
**SiAu**	-1.50~-1.90 (*vs.* RHE)	0.50~1.00	0.5 M $KHCO_3$	[10]
**H-E-MoS_2**	-0.40~-1.10 (*vs.* RHE)	0.25~2.00	EMIM-BF_4 aqueous	[11]
**r-Cu_{OX} (350)**	-0.25~-0.65 (*vs.* RHE)	1.00~3.00	0.1 M $KHCO_3$	[12]
**CF**	-0.50~-1.30 (*vs.* RHE)	0.50~3.00	0.1 M $KHCO_3$	[13]
**AgP_2**	-0.40~-1.00 (*vs.* RHE)	0.20~3.00	0.5 M $KHCO_3$	[14]
**3D hp CuAg**	-0.60~-1.00 (*vs.* RHE)	0.50~3.00	0.1 M $KHCO_3$	[15]

Table S3. Comparison of applications of other porous carbon-based catalysts.

Catalysis	Application	Ref
**BPN-3Dp-CCE**	**CO_2RR**	**This work**
**Porous 3-AF**	Super capacitor	[16]
**CCK-1**	Electrochemical Double Layer Capacitor (EDLC)	[17]
**Pt/CuO@C**	HER	[18]
**MoxC-NPC-1.0-800**	HER	[19]
**MoP@PC**	HER	[20]

[1] L. Meng, E. Zhang, H. Peng, Y. Wang, D. Wang, H. Rong, J. Zhang, Bi/Zn dual single-atom catalysts for electroreduction of CO_2 to syngas, ***ChemCatChem***, 14(7) (**2022**). https://doi.org/10.1002/cctc.202101801.

[2] H. Li, J. Gao, Q. Du, J. Shan, Y. Zhang, S. Wu, Z. Wang, Direct CO_2 electroreduction from NH_4HCO_3 electrolyte to syngas on bromine-modified Ag catalyst, ***Energy***, 216 (**2021**) 119250.

[3] Y.E. Kim, B. Kim, W. Lee, Y.N. Ko, M.H. Youn, S.K. Jeong, K.T. Park, J. Oh, Highly tunable syngas production by electrocatalytic reduction of CO_2 using Ag/TiO_2 catalysts, ***Chem. Eng. J.***, 413 (**2021**) 127448.

[4] C. Kim, S. Choi, M.-J. Choi, S.A. Lee, S.H. Ahn, S.Y. Kim, H.W. Jang, Photoelectrochemical reduction of CO_2 to syngas by reduced Ag catalysts on Si photocathodes, ***Appl. Sci.***, 10(10) (**2020**) 3487.

[5] X. Zhang, F.C. Romeiro, S.X. Guo, Y. Zhang, E. Nossol, R.C. Lima, A.M. Bond, J. Zhang, Size controllable metal nanoparticles anchored on nitrogen doped carbon for electrocatalytic energy conversion, ***Chemelectrochem***, 6(5) (**2019**) 1508-1513.

[6] R. He, A. Zhang, Y. Ding, T. Kong, Q. Xiao, H. Li, Y. Liu, J. Zeng, Achieving the widest range of syngas proportions at high current density over cadmium sulfoselenide nanorods in CO_2 electroreduction, ***Adv. Mater.***, 30(7) (**2018**) 1705872.

[7] Z. Geng, X. Kong, W. Chen, H. Su, Y. Liu, F. Cai, G. Wang, J. Zeng, Oxygen vacancies in ZnO nanosheets enhance CO_2 electrochemical reduction to CO, ***Angew.***

Chem. Int. Edit., 57(21) (**2018**) 6054-6059.

[8] R. Daiyan, R. Chen, P. Kumar, N.M. Bedford, J. Qu, J.M. Cairney, X. Lu, R. Amal, Tunable syngas production through CO_2 electroreduction on cbalt-carbon composite electrocatalyst, ***ACS Appl. Mater. Interfaces***, 12 (**2020**) 9307-9315.

[9] W.O. Silva, G.C. Silva, R.F. Webster, T.M. Benedetti, R.D. Tilley, E.A. Ticianelli, Electrochemical reduction of CO_2 on nitrogen-doped carbon catalysts with and without iron, ***Chemelectrochem***, 6(17) (**2019**) 4626-4636.

[10] M.B. Ross, C.T. Dinh, Y. Li, D. Kim, P. De Luna, E.H. Sargent, P. Yang, Tunable Cu enrichment enables designer syngas electrosynthesis from CO_2, ***J. Am. Chem. Soc.***, 139(27) (**2017**) 9359-9363.

[11] K. Lv, C. Teng, M. Shi, Y. Yuan, Y. Zhu, J. Wang, Z. Kong, X. Lu, Y. Zhu, Hydrophobic and electronic properties of the E-MoS_2 nanosheets induced by FAS for the CO_2 electroreduction to syngas with a wide range of CO/H_2 ratios, ***Adv. Funct. Mater.***, 28(49) (**2018**) 1802339.

[12] P. Chen, Y. Jiao, Y.-H. Zhu, S.-M. Chen, L. Song, M. Jaroniec, Y. Zheng, S.-Z. Qiao, Syngas production from electrocatalytic CO_2 reduction with high energetic efficiency and current density, ***J. Mater. Chem. A***, 7(13) (**2019**) 7675-7682.

[13] H. Li, N. Xiao, Y. Wang, C. Li, X. Ye, Z. Guo, X. Pan, C. Liu, J. Bai, J. Xiao, X. Zhang, S. Zhao, J. Qiu, Nitrogen-doped tubular carbon foam electrodes for efficient electroreduction of CO_2 to syngas with potential-independent CO/H_2 ratios, ***J. Mater. Chem. A***, 7(32) (**2019**) 18852-18860.

[14] H. Li, P. Wen, D.S. Itanze, Z.D. Hood, X. Ma, M. Kim, S. Adhikari, C. Lu, C. Dun, M. Chi, Y. Qiu, S.M. Geyer, Colloidal silver diphosphide (AgP_2) nanocrystals as low overpotential catalysts for CO_2 reduction to tunable syngas, ***Nat. Commun.*** 10(1) (**2019**) 5724.

[15] W.Y. Yan, C. Zhang, L. Liu, Hierarchically porous CuAg via 3D Printing/dealloying for tunable CO_2 reduction to syngas, ***ACS Appl. Mater. Inter.*** 13 (**2021**) 45385-45393.

[16] J.Y. Piao, D.S. Bin, S.Y. Duan, X.J. Lin, D. Zhang, A.M. Cao, A facile template free synthesis of porous carbon nanospheres with high capacitive performance, ***Sci. China. Chem.*** 61 (**2018**) 538-544.

[17] N. Sharma, N. Mishra, M. Sharon, M. Sharon, High performance supercapacitor using porous carbon nanomaterial from corn cob, National Conference on Carbon Materials (CCM) - Carbon Materials for Energy Harvesting, Environment, Nanoscience and Technology (Carbon Materials), Mumbai, INDIA, **2012**, pp. 219-223.

[18] Y. Dong, J. Ying, Y.X. Xiao, J.B. Chen, X.Y. Yang, Highly dispersed Pt nanoparticles Embedded in N-doped porous carbon for efficient hydrogen evolution, ***Chem. Asian J.*** 16 (**2021**) 1878-1881.

[19] Y.N. Zhang, D.Y. Kong, L.L. Bo, W.P. Shi, X.L. Guan, Y.X. Wang, Z.B. Lei, J.H. Tong, Electrospinning preparation of N, P dual-doped molybdenum carbide/porous carbon fibers with highly improved electrocatalytic activity for hydrogen evolution

reaction, ***ACS Appl. Energy Mater.*** 4 (**2021**) 13051-13060.

[20] J.S. Li, S. Zhang, J.Q. Sha, H. Wang, M.Z. Liu, L.X. Kong, G.D. Liu, Confined molybdenum phosphide in P-doped porous carbon as efficient electrocatalysts for hydrogen evolution, ***ACS Appl. Mater. Inter.*** 10 (**2018**) 17140-17146.
